# Assessment of the Correlation Between Obesity and Depression Among Adults in Saudi Arabia

**DOI:** 10.7759/cureus.74806

**Published:** 2024-11-30

**Authors:** Nasser Al Shanbari, Mohammed Basnawi, Ahmad O Bazarra, Hassan Khoj, Mohammed Alharhi, Bassil Almtrafi, Redwan Mirdad, Khalid Al-Zahrani, Mokhtar Shatla

**Affiliations:** 1 Internal Medicine, King Abdulaziz Medical City, Jeddah, SAU; 2 College of Medicine and Surgery, Umm Al-Qura University, Makkah, SAU; 3 Pharmacy, Saudi German Hospital, Makkah, SAU; 4 Dentistry, My Teeth &amp; My Beauty Medical Center, Baljurashi, SAU; 5 Pharmacy, Sukoon Long Term Care, Jeddah, SAU; 6 Pharmacy, Umm Al-Qura General Polyclinic, Makkah, SAU; 7 Community Medicine, Umm Al-Qura University, Makkah, SAU

**Keywords:** diet and depression, obesity complications, obesity medicine, obesity risk, risk of depression

## Abstract

Background

The prevalence of obesity has increased over the years, resulting in multiple physical and psychological health issues that impact the quality of human life. Numerous Western studies have linked obesity and depression, but few studies have investigated this correlation among the Saudi population. Hence, this study assesses the correlation between obesity and depression among the general population of Saudi Arabia.

Methods

To conduct this cross-sectional study, we used an electronic questionnaire obtained from previous studies and included the patient health questionnaire-9 (PHQ-9) to assess the study sample for depression. We enrolled 766 Saudi adults in our study. The data obtained from the participants was analyzed with IBM SPSS software using the chi-square test, Fisher’s exact test, ANOVA, and binary logistic regression.

Results

The majority of the participants were female (n = 391, 51%), aged between 18 and 25 years (n = 498, 65.0%). In terms of BMI, 18.3% were obese (n = 140). Regarding depressive symptoms assessment, 30% showed mild depression, making this the most prevalent category. Moderate depression was represented by 22.6% of the participants. The highest mean depressive symptom score was seen in the obese group (BMI ≥ 30.0), with a mean score of 10.04 (SD = 6.9).

Conclusion

A significant correlation was detected between the highest mean depressive symptom score and obesity, and psychological disorders or chronic disease also showed a significant association with depression.

## Introduction

Obesity and overweight are defined as the accumulation of excessive fat in various parts of the body, leading to multiple health issues that impact human life [1]. Several diseases result from obesity and overweight, including hypertension, diabetes, numerous cancers, and cardiovascular diseases [2]. Many countries suffer the consequences of the increasing prevalence of high BMI and its associated mortality. High BMI is associated with adverse health outcomes and places a financial burden on the population [3,4].

The incidence rate of obesity has dramatically increased, and it is now considered a worldwide health issue [5,6]. The rate of obesity in the Middle East (24.5%) is similar to the rates in European populations such as the United Kingdom (22.9%) and Germany (26.3%). The prevalence of obesity in Saudi Arabia is 23% (males: 23.1% and females: 24.2%) [7]. The high incidence of obesity in Middle Eastern countries is primarily caused by unhealthy behavioral habits, including a sedentary lifestyle, an unhealthy Westernized diet, lack of exercise, and other cultural and social factors [8].

In addition to the physical health impact, obesity may have psychological effects [9]. Western studies have shown a positive association between obesity and depression, with women more significantly associated with this relationship than men [10]. Research has also revealed that depression is positively associated with abdominal obesity, and some studies have demonstrated the causal relationship between depression and obesity [11,12].

In one Saudi study, mild to moderate symptoms of depression were found among the majority of the obese participants, and 41.7% of the depression cases were reported among obese individuals [13]. However, the association between obesity and depression has been investigated primarily in Western studies. There are limited studies of this association in the Saudi population, and the need for further research is highlighted by the impact of obesity on global public health, as established in the literature. This study addresses the research gap by investigating the presence of depressive symptoms among obese adults in Saudi Arabia.

## Materials and methods

Study design and ethical considerations

This cross-sectional study gathered data from the Saudi population using an electronic questionnaire made on Google Forms. The study was conducted in July 2024 after receiving ethical approval from the Biomedical Ethics Committee at the College of Medicine, Umm Al-Qura University, Makkah, Saudi Arabia (approval number: HAPO-02-K-012-2024-10-2218). The study was conducted according to the principles of the Declaration of Helsinki.

Study criteria

All adults aged 18 years or older living in Saudi Arabia were included in the study. However, non-Saudi residents and those who are using psychiatric medications were excluded due to their possible impact on the accuracy of the study scale results.

Sample size calculation

According to the size of the Saudi population reported by the Saudi General Authority for Statistics, we calculated our sample size to be at least 385 using Epi Info software v2.1 (CDC, Atlanta, Georgia), considering the confidence interval as 95% and the level of significance (p-value) as 5%. We distributed the survey to 822 individuals using a convenient sampling technique, and 766 met our inclusion criteria and agreed to participate.

Study tool

A structured self-administered online questionnaire was designed using a Google Drive questionnaire template with the latest updated version. The questions were derived from reviewed literature and previous studies [14,15]. Contact information of the corresponding author was added at the beginning of the survey to address any problems or questions, and study consent was obtained from the participants. The questionnaire was divided into two parts. The first part gathered participants' sociodemographic data and categorized respondents according to their BMI, and the second part contained the validated Arabic version of the Patient Health Questionnaire-9 (PHQ-9), which aims to assess depression symptoms among the participants [15].

Statistical analysis

A comprehensive statistical analysis of the dataset was conducted, encompassing descriptive and inferential methodologies. A descriptive analysis was performed to summarize participants’ demographic characteristics, which include age, gender, and other details. The chi-square test and Fisher’s exact test were used to determine the association between categorical variables. One-way ANOVA was used to establish the depression score differences between BMI categories. Subsequently, binary logistic regression was used to find the adjusted predictors for major depression. All statistical analyses were executed using IBM SPSS software, version 29 (IBM Corp., Armonk, NY).

## Results

The majority of participants were female (n = 391, 51%), aged between 18 and 25 years (n = 498, 65.0%). Most participants resided in the Eastern (n = 207, 27%) and Southern (n = 190, 24.8%) regions of Saudi Arabia. Participants’ marital status was predominantly single (n = 499, 65.1%), and the highest educational level achieved by most was an undergraduate degree (n = 517, 67.5%). A significant portion of participants had an average monthly income of ≤10,000 SAR (n = 506, 66.1%) and had five or fewer family members benefiting from this income (n = 464, 60.4%). Regarding BMI, 41.6% were classified as normal (n = 319), while 18.3% were obese (n = 140). The majority did not report any chronic disease (n = 679, 88.6%) or psychological disorders (n = 706, 92.2%) (Table 1).

**Table 1:** Sociodemographic and other parameters of participants (n = 766)

		Frequency, N (%)
Gender	Female	391 (51.0%)
	Male	375 (49.0%)
Age	18-25 years	498 (65.0%)
	26-30 years	43 (5.6%)
	31-35 years	39 (5.1%)
	36-40 years	37 (4.8%)
	41-45 years	49 (6.4%)
	46-50 years	57 (7.4%)
	>50 years	43 (5.6%)
Area of residence	Eastern	207 (27.0%)
	Southern	190 (24.8%)
	Central	166 (21.7%)
	Western	139 (18.1%)
	Northern	64 (8.4%)
Marital status	Single	499 (65.1%)
	Married	256 (33.4%)
	Divorced/Widowed	11 (1.4%)
Education		15 (2.0%)
	Secondary school	194 (25.3%)
	Undergraduate	517 (67.5%)
	Postgraduate	40 (5.2%)
Average monthly income	≤10,000 SAR	506 (66.1%)
	>10,000 SAR	260 (33.9%)
Number of family members benefiting from income	≤5	464 (60.6%)
	>5	302 (39.4%)
BMI	Underweight	106 (13.8%)
	Normal	319 (41.6%)
	Overweight	201 (26.2%)
	Obese	140 (18.3%)
Years with BMI ≥ 30 kg/m^2^	BMI is not 30/Don't know	535 (69.8%)
	<10 years	133 (17.4%)
	>10 years	98 (12.8%)
Chronic disease	No	679 (88.6%)
	Yes	87 (11.4%)
Diagnosed with psychological disorder	No	706 (92.2%)
	Yes	60 (7.8%)
Use psychiatric medicines	No	766 (100.0%)

Table 2 shows the assessment of participants’ depressive symptoms. A total of 206 participants (26.9%) never experienced a lack of interest or pleasure in doing things, while 352 (46.0%) experienced this on several days, and 83 (10.8%) experienced this on almost every day. Feeling upset or hopeless was absent in 223 participants (29.1%) but was present on several days in 363 participants (47.4%) and daily in 69 participants (9.0%). Sleep difficulties were not an issue for 237 participants (30.9%), but 259 (33.8%) faced them on several days, and 146 (19.1%) faced them almost daily. Feeling tired or inactive was reported by 351 participants (45.8%) on several days and 145 participants (18.9%) nearly every day. A total of 222 participants (29.0%) never experienced a lack of appetite or binge eating, while 280 participants (36.6%) suffered this on several days and 120 participants (15.7%) almost daily. Feelings of failure affected 89 participants (11.6%) almost daily, while difficulties concentrating troubled 81 participants (10.6%) every day. Moving, speaking slowly, or restlessness was reported by 198 participants (25.8%) on several days. Thoughts of dying or self-harm were present in 125 participants (16.3%) on several days and daily in 33 participants (4.3%).

**Table 2:** Assessment of depressive symptoms among participants (n = 766)

	Never	Several Days	Half of the Days	Almost Everyday
	N (%)	N (%)	N (%)	N (%)
Lack of interest or pleasure in doing things	206 (26.9%)	352 (46.0%)	125 (16.3%)	83 (10.8%)
Feeling upset, depressed, or hopeless	223 (29.1%)	363 (47.4%)	111 (14.5%)	69 (9.0%)
Difficulties in falling asleep, staying asleep, or sleeping too much	237 (30.9%)	259 (33.8%)	124 (16.2%)	146 (19.1%)
Feeling tired or lack of activity	123 (16.1%)	351 (45.8%)	147 (19.2%)	145 (18.9%)
Lack of appetite or binge eating	222 (29.0%)	280 (36.6%)	144 (18.8%)	120 (15.7%)
Feeling dissatisfied with yourself or feeling like a failure	376 (49.1%)	221 (28.9%)	80 (10.4%)	89 (11.6%)
Difficulties concentrating on things, such as reading the newspaper or watching television	358 (46.7%)	239 (31.2%)	88 (11.5%)	81 (10.6%)
Moving or speaking very slowly to the point of being noticeable or restlessness and inability to settle down	445 (58.1%)	198 (25.8%)	66 (8.6%)	57 (7.4%)
Thinking it would be better to die or thinking about harming oneself in some way	573 (74.8%)	125 (16.3%)	35 (4.6%)	33 (4.3%)

**Figure 1:**
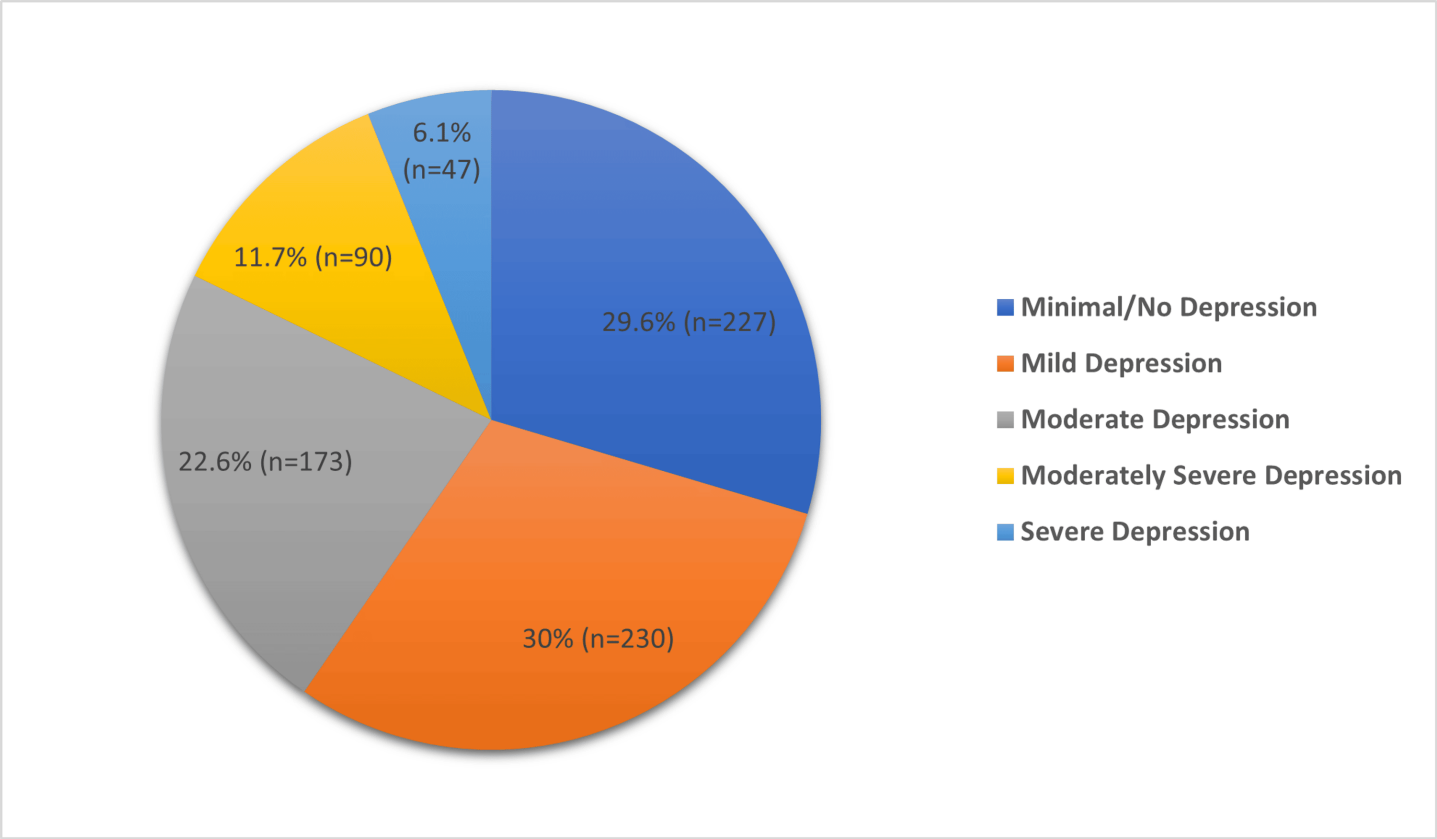
Overall depression level among participants based on PHQ-9 scale PHQ-9: Patient health questionnaire-9.

Figure 1 shows the distribution of depression levels among participants based on the PHQ-9 scale. It indicates that 29.6% of participants exhibit minimal or no depression, suggesting that nearly one-third of the population experiences little to no depressive symptoms. However, a substantial portion (30%) reports mild depression, making it the most prevalent category. Moderate depression affects 22.6% of the participants, indicating more pronounced symptoms. Moderately severe depression is observed in 11.7% of the participants, while severe depression, characterized by the most intense symptoms, affects 6.1% of the population.

**Figure 2:**
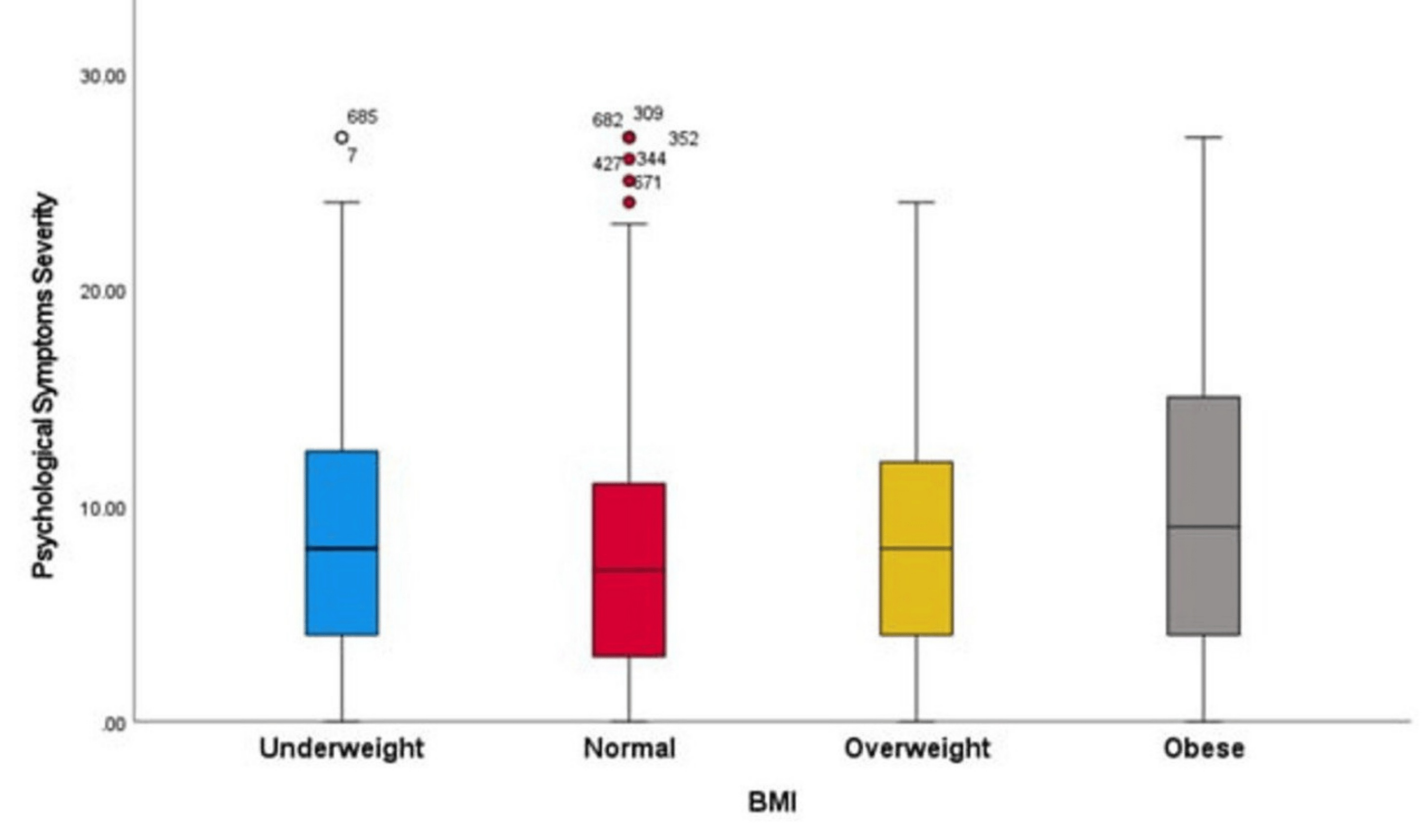
Boxplot showing depression score difference based on BMI level

Table 3 and Figure 2 show the differences in depressive symptom scores based on BMI categories among the 776 participants. The mean depressive symptom score was highest in the obese group (BMI ≥ 30.0), with a mean score of 10.04 (SD = 6.9). The underweight group (BMI < 18.5) had a mean score of 9.00 (SD = 6.1), and the overweight group (BMI = 25.0-29.9) had a mean score of 8.81 (SD = 5.7). Participants with a normal BMI (18.5-24.9) had the lowest mean depressive symptom score (8.10; SD = 5.8). The overall mean score for participants was 8.76 (SD = 6.1). A significant difference in depressive symptom scores across different BMI categories was observed, with a significant value of 0.017, indicating that BMI is significantly associated with the severity of depressive symptoms in this population.

**Table 3:** Difference of scores of depressive symptoms based on BMI (n = 776) ^a^ ANOVA. Note: p < 0.05 is significant.

	N	Mean (SD)	F Statistics	Sig. Value^a^
Underweight (<18.5)	107	9.00 (6.1)	3.399	0.017
Normal (18.5-24.9)	325	8.10 (5.8)		
Overweight (25.0-29.9)	203	8.81 (5.7)		
Obese (≥30.0)	141	10.04 (6.9)		
Total	776	8.76 (6.1)		

Table 4 shows the association between depression levels and various sociodemographic and health-related features using univariate analysis. Gender showed no significant association with depression levels, as females (60.1% minimal to mild and 39.9% moderate to severe) and males (59.7% minimal to mild and 40.3% moderate to severe) had similar distributions (p = 0.917). However, age was significantly associated with depression (p < 0.001), with younger participants (18-25 years) having a higher prevalence of moderate to severe depression (47.6%) than participants above 50 years old (20.9%). Area of residence also showed a significant association (p = 0.016), with participants from the Eastern region more likely to have minimal to mild depression (66.2%) than those from the Western region (53.2%). Marital status was another significant factor (p < 0.001), with single individuals having a higher prevalence of moderate to severe depression (47.5%) than married individuals (27.0%). Monthly income was significantly associated with depression levels (p = 0.018), with those earning less than 10,000 SAR more likely to have moderate to severe depression (43.1%) than those earning more (34.2%). Other factors, including education, number of family members, BMI, and chronic disease, did not show significant associations with depression levels. However, being diagnosed with a psychological disorder was strongly associated with higher levels of depression, with 80.0% of those diagnosed experiencing moderate to severe depression (p < 0.001).

**Table 4:** Association between depression level and different features including BMI (univariate analysis) ^a^ Chi-square test. ^b^ Fisher’s exact test. Note: p < 0.05 is significant.

		Depression		Chi-Square Statistics	Sig. Value
		Minimal to Mild (N, %)	Moderate to Severe (N, %)		
Gender	Female	235 (60.1%)	156 (39.9%)	0.011	0.917^a^
	Male	224 (59.7%)	151 (40.3%)		
Age	18-25 years	261 (52.4%)	237 (47.6%)	37.073	<0.001^a^
	26-30 years	28 (65.1%)	15 (34.9%)		
	31-35 years	26 (66.7%)	13 (33.3%)		
	36-40 years	29 (78.4%)	8 (21.6%)		
	41-45 years	36 (73.5%)	13 (26.5%)		
	46-50 years	45 (78.9%)	12 (21.1%)		
	>50 years	34 (79.1%)	9 (20.9%)		
Area of residence	Eastern	137 (66.2%)	70 (33.8%)	12.242	0.016^a^
	Southern	124 (65.3%)	66 (34.7%)		
	Central	87 (52.4%)	79 (47.6%)		
	Western	74 (53.2%)	65 (46.8%)		
	Northern	37 (57.8%)	27 (42.2%)		
Marital status	Divorced/Widowed	10 (90.9%)	1 (9.1%)	34.191	<0.001^b^
	Single	262 (52.5%)	237 (47.5%)		
	Married	187 (73.0%)	69 (27.0%)		
Education		10 (66.7%)	5 (33.3%)	0.563	0.905^a^
	Secondary school	119 (61.3%)	75 (38.7%)		
	Undergraduate	306 (59.2%)	211 (40.8%)		
	Postgraduate	24 (60.0%)	16 (40.0%)		
Monthly income	<10,000 SAR	288 (56.9%)	218 (43.1%)	5.604	0.018^a^
	>10,000 SAR	171 (65.8%)	89 (34.2%)		
Number of family members	≤5	272 (58.6%)	192 (41.4%)	0.829	0.362^a^
	>5	187 (61.9%)	115 (38.1%)		
BMI	Underweight	63 (59.4%)	43 (40.6%)	6.165	0.104^a^
	Normal	205 (64.3%)	114 (35.7%)		
	Overweight	118 (58.7%)	83 (41.3%)		
	Obese	73 (52.1%)	67 (47.9%)		
Years with a BMI ≥ 30 kg^2^	BMI is not 30/Don't know	322 (60.2%)	213 (39.8%)	3.544	0.170^a^
	<10 years	72 (54.1%)	61 (45.9%)		
	>10 years	65 (66.3%)	33 (33.7%)		
Chronic disease	No	414 (61.0%)	265 (39.0%)	2.746	0.097^a^
	Yes	45 (51.7%)	42 (48.3%)		
Diagnosed with psychological disorders	No	447 (63.3%)	259 (36.7%)	43.202	<0.001^a^
	Yes	12 (20.0%)	48 (80.0%)		

Table 5 shows the results of the multivariate analysis, identifying the adjusted predictors of severe and major depression. Gender was not a significant predictor, with males showing no strong evidence of increased risk (p = 0.175, OR = 0.779). However, age was a significant negative predictor (p < 0.001, OR = 0.738), indicating that older participants were less likely to experience severe depression, and younger patients were more likely to have severe depression. BMI and the number of years with a BMI of 30 kg/m² or more did not show significant associations with depression severity (p = 0.202 and p = 0.707, respectively). Area of residence and marital status were not significant predictors (p = 0.546 and p = 0.782, respectively). Higher education did not significantly influence the likelihood of severe depression (p = 0.949, OR = 1.011). However, monthly income over 10,000 SAR was associated with a higher likelihood of severe depression (p < 0.001, OR = 1.397). Chronic disease emerged as a significant predictor, with those having a chronic disease being likelier to suffer from severe depression (p = 0.016, OR = 1.886). The presence of a psychological disorder was the strongest predictor, showing a very high association with severe depression (p < 0.001, OR = 6.682).

**Table 5:** Adjusted predictors of severe and major depression (multivariate analysis) Binary logistic regression analysis. Exp (B): Odd’s ratio. Note: p < 0.05 is significant.

	B	Sig.	Exp (B)	95% CI	
				Lower	Upper
Gender (Male)	-.250	.175	.779	.543	1.117
Age (Years)	-.304	.000	.738	.645	.845
BMI	.091	.202	1.095	.952	1.260
No. of years with BMI ≥ 30 kg^2^	-.091	.707	.913	.567	1.469
Area of residence	.086	.546	1.090	.824	1.441
Marital status (married)	.052	.782	1.053	.728	1.524
Higher education	.011	.949	1.011	.728	1.403
Monthly income (>10,000 SAR)	.334	.000	1.397	1.167	1.673
No. of family members benefit from income	-.045	.706	.956	.756	1.209
Chronic disease (Yes)	.634	.016	1.886	1.125	3.161
Psychological disorders (Yes)	1.899	.000	6.682	3.375	13.229
Constant	-1.052	.062	.349		

## Discussion

Multiple Western studies have demonstrated an association between obesity and depression [10]. This associated results from the conditions’ shared biological pathways and the intersecting pathophysiology that eventually negatively impacts health and quality of life [16,17].

We assessed the correlation between obesity and depression among the Saudi general population. The chi-square test did not show a significant association between obesity and depression in the study. However, the highest mean depressive symptom scores were reported by obese, underweight, and overweight participants, respectively. Hence, abnormal alterations in BMI levels correlate with increased severity of depressive symptoms. Similarly, most depression cases reported in a study conducted in Jeddah were found among overweight (19.5%) and obese (41.7%) individuals [13]. Moreover, a study conducted in the eastern province of Saudi Arabia found a significant association, with 41.7% of obese participants showing moderate to severe depression [14].

An Australian study conflicted with these findings, reporting lower percentages of depression among overweight (12%) and obese (23%) participants [18]. The variations of the significance and the degree of association between obesity and depression among different populations could be due to not excluding those who use psychiatric and non-psychiatric medications that could be associated with variability in the severity of depressive symptoms and alterations in body weight [19,20]. Therefore, future studies should ensure precise methodology, especially regarding inclusion and exclusion criteria, to enable researchers to investigate the causal relationship between obesity and depression.

Other factors could contribute to the increase in depression incidence. The present study found no statistically significant association between depression and chronic diseases. However, the multivariate analysis revealed that chronic disease was a significant predictor, as individuals with chronic disease are more likely to experience severe depressive symptoms. Significant associations were detected between hypertension, diabetes mellitus, and depression [13]. A previous study revealed a significant association between diabetes, hypertension, obesity, and depression [21]. These findings necessitate further investigations to identify and analyze these factors, as they could increase depression prevalence among the Saudi population.

A recent systematic review found that more than one-third of Saudi adults suffer from depression [22]. In our study, 30% of the survey respondents had mild depression, and 22.6% had moderate depression, whereas only 29.6% of the participants showed minimal or no depression. These findings align with a study that reported moderate to severe depression among 29.7% of participants [13]. Additionally, a prior study observed that 34.8% of participants suffered moderate to severe depression [14].

Moreover, 18.3% of our respondents were obese, and 26.2% were overweight. A similar study also reported figures of 26.4% obese and 30.5% overweight [14], and another Saudi study found 38.4% obesity and 44.2% overweight prevalence [23]. Henceforth, interventions that alleviate obesity should be encouraged with the aim of improving mental health and decreasing the prevalence of depression among the Saudi population.

Strengths and limitations

To the best of our knowledge, this is the first nationwide survey in Saudi Arabia to include participants from all the country's provinces and use multiple statistical and analytical tests to look for correlations between obesity and depression. Nevertheless, the study could be limited by possible recall bias since the study questionnaire was self-reported. Moreover, the randomization of the study sample could be affected due to the sampling technique used. Therefore, further studies with more stringent methodologies and more randomized sampling techniques are needed to support the evidence of the existence of the association between obesity and depression and to identify the causal relationship between them.

## Conclusions

The impetus for this study arose from the growing public health concern of the co-occurrence of obesity and depression. We assessed the correlation between obesity and depression among the Saudi population, finding a significant correlation between obesity and the highest mean depressive score. Other factors were significantly associated with depression, including having a psychological disorder and suffering from chronic disease. Further studies using more stringent methodologies and a wider sample size are recommended to support the current evidence of the association between obesity and depression and to enhance interventions that improve BMI in an attempt to mitigate the severity of depressive symptoms and improve mental health.

## References

[1] Abdelaal M, le Roux CW, Docherty NG (2017). Morbidity and mortality associated with obesity. Ann Transl Med.

[2] Guh DP, Zhang W, Bansback N, Amarsi Z, Birmingham CL, Anis AH (2009). The incidence of co-morbidities related to obesity and overweight: a systematic review and meta-analysis. BMC Public Health.

[3] Dai H, Alsalhe TA, Chalghaf N, Riccò M, Bragazzi NL, Wu J (2020). The global burden of disease attributable to high body mass index in 195 countries and territories, 1990-2017: an analysis of the Global Burden of Disease Study. PLoS Med.

[4] Tremmel M, Gerdtham UG, Nilsson PM, Saha S (2017). Economic burden of obesity: a systematic literature review. Int J Environ Res Public Health.

[5] Kelly T, Yang W, Chen CS, Reynolds K, He J (2008). Global burden of obesity in 2005 and projections to 2030. Int J Obes (Lond).

[6] Low S, Chin MC, Deurenberg-Yap M (2009). Review on epidemic of obesity. Ann Acad Med Singap.

[7] Alsulami S, Baig M, Ahmad T (2023). Obesity prevalence, physical activity, and dietary practices among adults in Saudi Arabia. Front Public Health.

[8] Musaiger AO (2011). Overweight and obesity in eastern mediterranean region: prevalence and possible causes. J Obes.

[9] Ackner SE (2005). The obese patient: the psychosocial burden of obesity and the role of bariatric surgery. Psychiatry (Edgmont).

[10] de Wit L, Luppino F, van Straten A, Penninx B, Zitman F, Cuijpers P (2010). Depression and obesity: a meta-analysis of community-based studies. Psychiatry Res.

[11] Xu Q, Anderson D, Lurie-Beck J (2011). The relationship between abdominal obesity and depression in the general population: a systematic review and meta-analysis. Obes Res Clin Pract.

[12] Luppino FS, de Wit LM, Bouvy PF, Stijnen T, Cuijpers P, Penninx BW, Zitman FG (2010). Overweight, obesity, and depression: a systematic review and meta-analysis of longitudinal studies. Arch Gen Psychiatry.

[13] Alkharji TM, Alharbi RS, Bakhsh EA, Alghalibi M, Alraddadi RA (2023). The association between depression and obesity among adults in Jeddah, Saudi Arabia, in 2022. Cureus.

[14] Almarhoon FH, Almubarak KA, Alramdhan ZA, Albagshi RS, Alotayriz JK, Alqahtani AH (2021). The association between depression and obesity among adults in the Eastern Province, Saudi Arabia. Cureus.

[15] AlHadi AN, AlAteeq DA, Al-Sharif E (2017). An arabic translation, reliability, and validation of Patient Health Questionnaire in a Saudi sample. Ann Gen Psychiatry.

[16] Jantaratnotai N, Mosikanon K, Lee Y, McIntyre RS (2017). The interface of depression and obesity. Obes Res Clin Pract.

[17] Milaneschi Y, Simmons WK, van Rossum EF, Penninx BW (2019). Depression and obesity: evidence of shared biological mechanisms. Mol Psychiatry.

[18] Carey M, Small H, Yoong SL, Boyes A, Bisquera A, Sanson-Fisher R (2014). Prevalence of comorbid depression and obesity in general practice: a cross-sectional survey. Br J Gen Pract.

[19] Celano CM, Freudenreich O, Fernandez-Robles C, Stern TA, Caro MA, Huffman JC (2011). Depressogenic effects of medications: a review. Dialogues Clin Neurosci.

[20] Verhaegen AA, Van Gaal LF (2021). Drugs affecting body weight, body fat distribution, and metabolic function-mechanisms and possible therapeutic or preventive measures: an update. Curr Obes Rep.

[21] Stecker T, Fortney JC, Steffick DE, Prajapati S (2006). The triple threat for chronic disease: obesity, race, and depression. Psychosomatics.

[22] Nour MO, Alharbi KK, Hafiz TA (2023). Prevalence of depression and associated factors among adults in Saudi Arabia: systematic review and meta-analysis (2000-2022). Depress Anxiety.

[23] Alshahrani MMA, Al-Masoudi M, Alshahrani EM (2021). Association between obesity and mental disorders among male secondary school students in Abha, Kingdom of Saudi Arabia: a predictor based cross-sectional study. World Fam Med J/Middle East J Fam Med.

